# Comparative peculiarities of genomic diversity in Gallus gallus domesticus chickens with decorative plumage: the muffs and beard phenotype

**DOI:** 10.18699/vjgb-24-13

**Published:** 2024-02

**Authors:** N.V. Dementieva, Y.S. Shcherbakov, A.E. Ryabova, A.B. Vakhrameev, A.V. Makarova, O.A. Nikolaeva, A.P. Dysin, A.I. Azovtseva, N.R. Reinbah, O.V. Mitrofanova

**Affiliations:** Russian Research Institute of Farm Animal Genetics and Breeding – Branch of the L.K. Ernst Federal Research Center for Animal Husbandry, Tyarlevo, St. Petersburg, Russia; Russian Research Institute of Farm Animal Genetics and Breeding – Branch of the L.K. Ernst Federal Research Center for Animal Husbandry, Tyarlevo, St. Petersburg, Russia; Russian Research Institute of Farm Animal Genetics and Breeding – Branch of the L.K. Ernst Federal Research Center for Animal Husbandry, Tyarlevo, St. Petersburg, Russia; Russian Research Institute of Farm Animal Genetics and Breeding – Branch of the L.K. Ernst Federal Research Center for Animal Husbandry, Tyarlevo, St. Petersburg, Russia; Russian Research Institute of Farm Animal Genetics and Breeding – Branch of the L.K. Ernst Federal Research Center for Animal Husbandry, Tyarlevo, St. Petersburg, Russia; Russian Research Institute of Farm Animal Genetics and Breeding – Branch of the L.K. Ernst Federal Research Center for Animal Husbandry, Tyarlevo, St. Petersburg, Russia; Russian Research Institute of Farm Animal Genetics and Breeding – Branch of the L.K. Ernst Federal Research Center for Animal Husbandry, Tyarlevo, St. Petersburg, Russia; Russian Research Institute of Farm Animal Genetics and Breeding – Branch of the L.K. Ernst Federal Research Center for Animal Husbandry, Tyarlevo, St. Petersburg, Russia; Russian Research Institute of Farm Animal Genetics and Breeding – Branch of the L.K. Ernst Federal Research Center for Animal Husbandry, Tyarlevo, St. Petersburg, Russia; Russian Research Institute of Farm Animal Genetics and Breeding – Branch of the L.K. Ernst Federal Research Center for Animal Husbandry, Tyarlevo, St. Petersburg, Russia

**Keywords:** whole-genome genotyping, SNP marker, phenotype, genotype, genetic diversity, polymorphism, heterozygosity, DNA, chicken breeds, полногеномное генотипирование, SNP-маркер, фенотип, генотип, генетическое разнообразие, полиморфизм, гетерозиготность, ДНК, породы кур

## Abstract

Throughout history, humans have been attempting to develop the ornamental features of domestic animals in addition to their productive qualities. Many chicken breeds have developed tufts of elongated feathers that jut out from the sides and bottom of the beak, leading to the phenotype known as muffs and beard. It is an incomplete autosomal dominant phenotype determined by the Mb locus localised on chromosome GGA27. This project aimed to analyse the genetic diversity of chicken breeds using full genomic genotyping with the Chicken 60K BeadChip. A total of 53,313 Single Nucleotide Polymorphisms were analysed. DNA was obtained from breeds with the muffs and beard as a marker phenotype: Faverolles (n = 20), Ukrainian Muffed (n = 18), Orloff (n = 20), Novopavlov White (n = 20), and Novopavlov Coloured (n = 15). The Russian White (n = 20) was selected as an alternative breed without the muffs and beard phenotype. The chickens are owned by the Centre of Collective Use “Genetic Collection of Rare and Endangered Breeds of Chickens” (St. Petersburg region, Pushkin), and are also included in the Core Shared Research Facility (CSRF) and/or Large-Scale Research Facility (LSRF). Multidimensional scaling revealed that the Novopavlov White and the Novopavlov Coloured populations formed a separate group. The Ukrainian Muffed and the Orloff have also been combined into a separate group. Based on cluster analysis, with the cross-validation error and the most probable number of clusters K = 4 taken into account, the Orloff was singled out as a separate group. The Ukrainian Muffed exhibited a notable similarity with the Orloff under the same conditions. At K = 5, the populations of the Novopavlov White and the Novopavlov Coloured diverged. Only at K = 6, a distinct and separate cluster was formed by the Ukrainian Muffed. The Russian White had the greatest number of short (1–2 Mb) homozygous regions. If the HOXB8 gene is located between 3.402 and 3.404 Mb on chromosome GGA27, homozygous regions are rarely found in the chickens with the muffs and beard phenotype. Scanning the chicken genome with the Chicken 60K BeadChip provided enough information about the genetic diversity of the chicken breeds for the peculiarities of the development of the ornamental muffs and beard phenotypes in them to be understood. For example, Phoenix bantams, whose tail feathers grow throughout their lives, require greater consideration of husbandry conditions.

## Abstract (ru)

На протяжении истории взаимодействия с домашними животными человек стремился усилить не только их продуктивные качества, но и различные декоративные особенности. У кур ряда пород сформировались пучки удлиненных перьев, выступающих сбоку и снизу от клюва, образуя фенотип, описываемый как «баки и борода» (англ. muffs and beard). Это неполный аутосомно-доминантный фенотип, кодируемый локусом Mb, локализованным на хромосоме GGA27. Цель нашей работы – проанализировать генетическое разнообразие пород кур, определенное с помощью полногеномного генотипирования с использованием чипов Chicken 60K BeadChip. Всего в анализе учитывалось 53 313 однонуклеотидных полиморфных замен (SNP). ДНК получена от пород, обладающих маркерным признаком «баки и борода»: фавероль (n = 20), украинская ушанка (n = 18), орловская (n = 20), новопавловская белая (n = 20) и новопавловская цветная (n = 15). В качестве альтернативной породы, не имеющей фенотипа «баки и борода», использовалась русская белая (n = 20). Птица содержалась в ЦКП «Генетическая коллекция редких и исчезающих пород кур» (г. Санкт-Петербург, Пушкин), входящем в состав Сетевой биоресурсной коллекции животных и птиц. Методом многомерного шкалирования установлено, что отдельную группировку образовали популяции новопавловская белая и новопавловская цветная. В самостоятельную группу объединились породы украинская ушанка и орловская. С помощью кластерного анализа с учетом ошибки кросс-валидации и наиболее вероятным числом кластеров K = 4 орловская порода выделена в отдельную группу. Украинская ушанка в этом случае продемонстрировала значительное сходство с орловской породой. При K = 5 разделились новопавловская белая и новопавловская цветная популяции. И только при K = 6 явный отдельный кластер образовала украинская ушанка. У кур русской белой породы отмечено наибольшее количество коротких (1–2 Mб) гомозиготных районов. В месте расположения гена HOXB8 в регионе 3.402–3.404 Мб на хромосоме GGA27 у представителей пород c фенотипом «баки и борода» гомозиготные районы встречаются редко. Сканирование генома кур с использованием чипа Chicken 60K BeadChip позволяет получить достаточно информации о генетическом разнообразии пород кур для понимания особенностей формирования у них декоративного фенотипа «баки и борода».

## Introduction

The domestic chicken (Gallus gallus domesticus) is one of the most widespread domesticated animals in the world. This species assumes a prominent role in human society, providing the largest source of animal protein, as well as being an important factor in sociocultural development (Lawal, Hanotte, 2021). Since becoming domesticated, chickens have spread to different countries and continents, resulting in the numerous breeds we know today.

Genetic variability is a key part of the study of evolution, development and differentiation of living organisms. In domestic animals, breeds are organised into a specific system that evolves in accordance with the tasks defined by humans. As a result, there are remarkable phenotypes that distinguish domesticated animals from their wild ancestors

Throughout history, humans have sought to improve not only the production of animals, but their ornamentation as well. Such features, when severe, can have a negative impact on the lives of individuals who possess them. However, a significant proportion of the ornamental traits common to different breeds of chickens have no negative effect on the animals. Some chicken breeds may exhibit morphological traits characterized by elongated feathers growing from the sides and bottom of the beak, creating a distinctive phenotype called “muffs and beard”. It is an incomplete autosomal dominant phenotype encoded by the Mb locus

The feathers surrounding the beard and muffs in chickens vary considerably in shape and length. Certain breeds have a voluminous muff from ear to ear (Novopavlov chickens, Houndan, Crèvecoeur, etc.), others have a weak expression of it. In certain instances, such as in the case of Barbu d’Anvers chickens, it is mainly the muffs that are developed, and the throat part of the beard is barely visible.

When studying the genomic characteristics of chickens with the “muffs and beard” phenotype, it was proposed that the Mb allele is localised on GGA27, forming a complex structural variation in the genome that leads to altered expression of the HOXB8 gene (Guo Y. et al., 2016). Other researchers have also found regions implicated in the development of this trait by using whole-genome analysis of GGA1, GGA2 and GGA27. The analysis of the HOXB8 gene family members showed that it had different evolutionary dynamics among animals and that its motifs were conserved among avians, reptiles, amphibians and mammals, except for fish, whose HOXB8 protein lost motif 10. This suggests a potential role for HOXB8 in the evolution of sophisticated skin structures such as keratinous appendages. The authors highlight the intricate protein interactions of the HOXB family gene products in chickens, which are thought to contribute to understanding of the mechanisms of development and differentiation of the “muffs and beard” phenotype (Yang et al., 2020).

There is currently limited information concerning the regulation of head feathering in chickens due to the phenotypic diversity of the muff and beard traits. Of particular significance is the necessity for employing gene pool breeds as model objects in the search for new candidate genes, for example ones associated with hair growth in humans and animals. Selection without a strict focus on productive traits enables the attainment of a greater genetic diversity in the gene pool of chicken breeds, unlike that of industrial populations. Therefore, the study of their genomes can provide new information about structural changes in genes, the accumulation of homozygous regions and other genetic features.

**Fig. 1.**
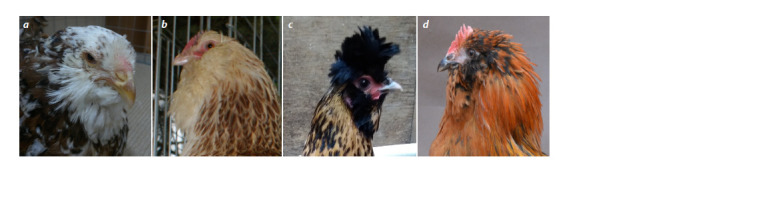
Expression of the “muffs and beard” phenotype in chickens of different breeds: a – Orloff; b – Faverolles; c – Novopavlov; d – Ukrainian Muffed.

Genetic Collection of Rare and Endangered Chicken Breeds, which is a part of Core Shared Research Facility (CSRF) and/ or Large-Scale Research Facility (LSRF), contains several breeds with this trait: Faverolles, Ukrainian Muffed, Orloff, and Novopavlov (Paronyan et al., 2016). Orloff Mille Fleur is a historic Russian chicken breed with distinct physical characteristics and a manifest “muffs and beard” trait. The Ukrainian Muffed belongs to local heritage breeds of the southern regions of Ukraine and Russia, and has a variety of plumage colours. Faverolles, a historic French breed, is renowned for its rich and delectable meat. Additionally, it possesses voluminous ornamental muffs and beard. The Novopavlov breed is a phenotypically restored ancient ornamental breed of Pavlovo chickens, distinguished by their muffs and beard (Fig. 1). Furthermore, the Centre of Collective Use also includes breeds that do not have the Mb allele in their genome

The purpose of our study was to examine the genetic variation of chicken breeds harboring the genetic trait “muffs and beard” via whole-genome genotyping with the Chicken 60K BeadChip, in order to obtain new information on structural changes in the genomes, accumulation of homozygous regions and other genetic characteristics

## Materials and methods

The material of the study was genomic DNA of chickens held in the bioresource collection of the Russian Research Institute of Farm Animal Genetics and Breeding – Branch of the L.K. Ernst Federal Research Centre for Animal Husbandry “Genetic Collection of Rare and Endangered Breeds of Chickens” (St. Petersburg-Pushkin), which is part of the Core Shared Research Facility (CSRF) and/or Large-Scale Research Facility (LSRF).

For this study, chickens were selected with the marker traits “muffs and beard”. These were Faverolles (n = 20), Ukrainian Muffed (n = 18), Orloff (n = 20), along with representatives from two groups of Novopavlov chickens: Novopavlov White (n = 20) and Novopavlov Coloured (n = 15). As an alternative breed without the “muffs and beard” phenotype, the Russian White breed was chosen (n = 20).

Chicken blood was collected from the axillary vein into microtubes containing 30 μl 0.5 M EDTA anticoagulant. Genomic DNA was extracted by phenol-chloroform extraction

To assess the purity of isolated DNA, its quality was determined by measuring the absorbance at 260 and 280 nm (OD260/280) using a NanoDrop 2000 instrument (Thermo Fisher Scientific Inc., Waltham, MA, USA) in accordance with the manufacturer’s instructions. DNA samples with OD260/280 values between 1.6 and 2.0 were chosen for whole-genome genotyping.

Whole-genome genotyping was performed with a medium density Chicken 60K BeadChip (Illumina Inc., USA) DNA chip containing ~50,000 SNPs. The acquired whole-genome data facilitated the evaluation of genetic diversity through DNA sequence polymorphism analysis. A comprehensive analysis of 53,313 SNPs was conducted.

Quality control and filtering of genotyping data for each SNP and each sample were carried out using the PLINK 1.9 software package (http://zzz.bwh.harvard.edu/plink). Sample genotyping quality was assessed using the following filters: the proportion of genotyped SNPs out of the total number of SNPs on the DNA chip for each tested SNP in an individual sample was greater than 90 %; the genotyping quality for each tested SNP across all genotyped samples was also greater than 90 %; the frequency of minor allele occurrence was greater than 1 %; and the deviation of SNP genotypes from the Hardy–Weinberg equilibrium probability was less than 10–6.

To evaluate the genetic diversity, the following indices were calculated using the R package diveRsity (Keenan et al., 2013): HO for observed heterozygosity, HE for expected heterozygosity, F for inbreeding coefficient, and Fmin and Fmax for minimum and maximum detected inbreeding coefficients, respectively

In order to assess the structure of the genome of the various breeds, we used the method of analysing the number and average length of runs of homozygosity (ROHs), the method of multidimensional scaling (MDS) based on the identityby- state (IBS) matrix, and the method of FST analysis of the genetic divergence of populations

A method for detecting ROHs was employed using sequential SNP detection, through the R package detectRUNS (Biscarini et al., 2018). To avoid underestimating the quantity of ROHs exceeding 8 Mb in length, we permitted only one SNP with an absent genotype and no more than one probable heterozygous genotype (Ferenčaković et al., 2013). To prevent common ROHs, the minimum ROH length was set as 1 Mb.

To minimise the number of false-positive results, the minimum number of SNPs was calculated as follows (Purfield et al., 2012):

**Formula. 1.**
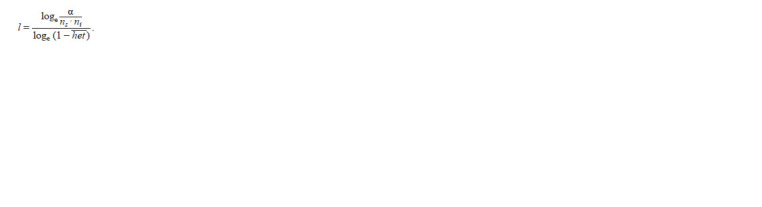
Formula 1

Where l is the minimum number of SNPs forming ROHs, ns is the number of SNPs per individual, ni is the number of genotyped individuals, α is the false-positive rate of ROHs (set to 0.05) and het is the mean heterozygosity of the total SNPs within population. Here, a minimum of 23 SNPs was found.

Initially, the number and length of ROHs were determined for each individual, and then their mean values within each breed were computed. Additionally, the ROH-based genomic inbreeding coefficient (FROH) was calculated as the ratio of the sum of the length of all ROHs per animal to the total length of the autosomal genome. Then, the number of ROHs in the genome of the breeds under study was determined by length classes: (1, 2), (2, 4), (4, 8), (8, 16) and >16 Mb. To establish the genome proportion that is overlapped by various ROH segments, we added up ROHs for categories that possess diverse minimum lengths (>1, >2, >4, >8, and >16 Mb).

MDS was conducted in PLINK 1.9 (Anderson et al., 2010), followed by plotting in the R package ggplot2. The fixation index FST was calculated using the EIGENSOFT 6.1.4 (Price et al., 2006) package with graphical representation performed in the SplitsTree software (Huson, Bryant, 2006).

We assessed the genetic structure of the analysed breeds in the Admixture 1.3 (Alexander et al., 2009) software and visually displayed it using the R package Pophelper (Francis, 2017). The optimal number of clusters (K) was determined by using the most likely number of ancestral clusters and calculating cross-validation error (CV error) values in the Admixture 1.3 software

The phylogenetic tree of the studied chicken populations was constructed using the Neighbor-Net (Bryant, Moulton, 2004) method in the iTOL service (Letunic, Bork, 2021) based on pairwise genetic distances FST.

The pairwise genetic distances FST were estimated using the R package StaMPP (Pembleton et al., 2013). The significance of the obtained results (P) was calculated based on the analysis of 100 permutations

## Results

Indicators of genetic diversity in the studied breeds are presented in Table 1.

**Table 1.**
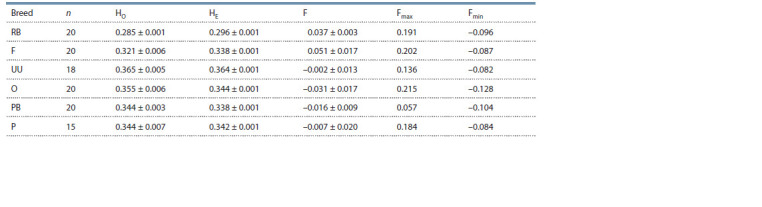
Genetic diversity in the studied breeds n – number of individuals in the analysis; HO – observed heterozygosity; HE – expected heterozygosity; F – inbreeding coefficient; Fmax – maximum detected inbreeding coefficient; Fmin – minimum detected inbreeding coefficient. At α = 0.05, there is no statistically significant difference. RB – Russian White, F – Faverolles, UU – Ukrainian Muffed, O – Orloff, PB – Novopavlov (White population), P – Novopavlov (Coloured population).

**Fig. 2.**
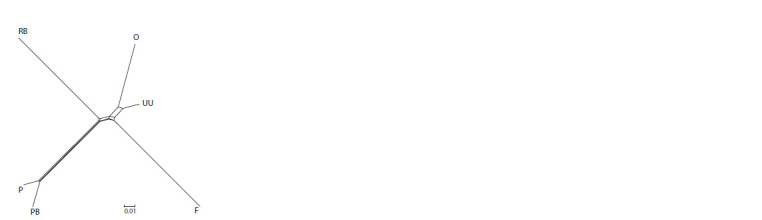
Phylogenetic tree of the studied chicken populations based on pairwise genetic distances FST, constructed with the Neighbor-Net method. RB – Russian White, F – Faverolles, UU – Ukrainian Muffed, O – Orloff, PB – Novopavlov (White population), P – Novopavlov (Coloured population).

Pairwise genetic distances FST, as shown in Table 2, ranged from 0.037 (Novopavlov Coloured and Novopavlov White) to 0.220 (between Russian White and Faverolles). The FST value for the identified cluster consisting of Ukrainian Muffed and Orloff was 0.075. A visual representation of the FST genetic distances is given in Figure 2.

**Table 2.**
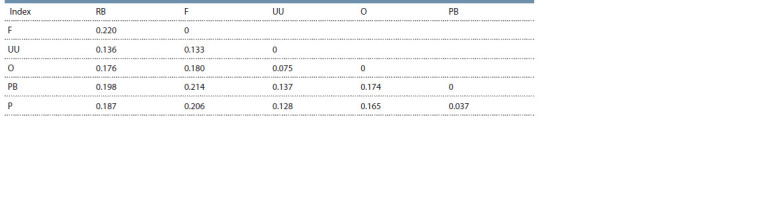
Pairwise genetic distances of FST between the studied chicken breeds RB – Russian White, F – Faverolles, UU – Ukrainian Muffed, O – Orloff, PB – Novopavlov (White population), P – Novopavlov (Coloured population). The statistical significance for all pairs of comparisons is set at p < 0.0001.

**Fig. 3.**
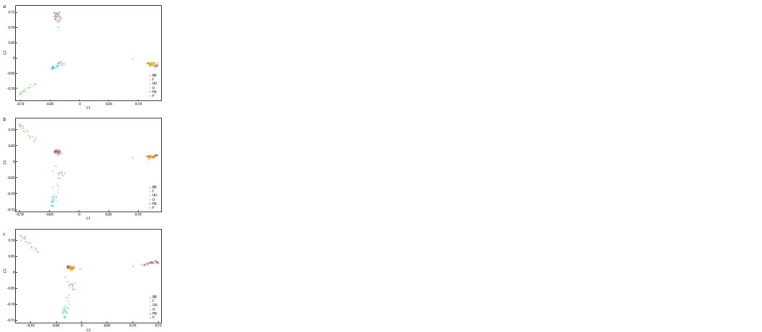
Multidimensional scaling graph. RB – Russian White, F – Faverolles, UU – Ukrainian Muffed, O – Orloff, PB – Novopavlov (White population), P – Novopavlov (Coloured population).

Figure 3 shows the results of the MDS analysis visualising the data. This approach makes it possible to analyse and visualise the points corresponding to the objects of interest in a way that minimises the distance between them. The objects are plotted in the diagram based on the selected principal component system. The results of MDS indicated that the Novopavlov White and Novopavlov Coloured populations formed a distinct cluster, whereas the Ukrainian Muffed and Orloff chicken breeds formed a separate cluster. There was no change in clustering when comparing the various components.

**Fig. 4.**
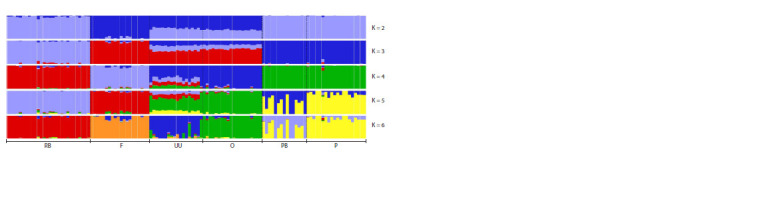
Admixture cluster analysis conducted for six chicken breeds using whole-genome SNP analysis. RB – Russian White, F – Faverolles, UU – Ukrainian Muffed, O – Orloff, PB – Novopavlov (White population), P – Novopavlov (Coloured population).

The CV error calculation in Admixture cluster analysis indicated that there are likely four clusters (K) in our sample. The cross-validation error was the lowest in this case (CV error = 0.54930). According to the admixture analysis (Fig. 4), the Orloff breed was represented as a separate cluster at K = 4. The Ukrainian Muffed comprises of a genetically and phenotypically identical population that demonstrates noteworthy similarities with the Orloff breed. At K = 5, the populations of white and coloured individuals in Novopavlov chickens were separated. At K = 6, a distinct cluster was formed by the Ukrainian Muffed breed, which contained individuals with similar genetic structures to the Orloff breed

**Fig. 5.**
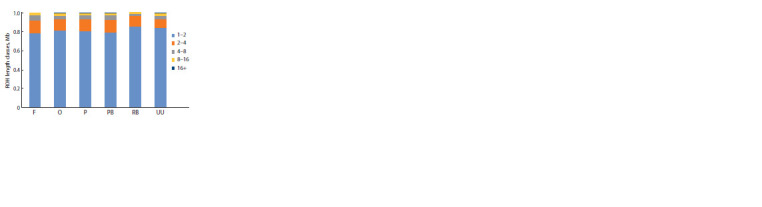
Distribution of the number of runs of homozygosity (ROHs) in relation to their average length in the studied groups of chickens. RB – Russian White, F – Faverolles, UU – Ukrainian Muffed, O – Orloff, PB – Novopavlov (White population), P – Novopavlov (Coloured population).

Analysis of the distribution of homozygous regions by length showed that the Russian White breed had the largest number of short homozygous regions (1–2 Mb), whereas the Faverolles breed had the smallest number of short homozygous regions (Fig. 5). Homozygous regions of class 16+ were more prevalent in the coloured population of the Novopavlov breed and the least prevalent in the Russian White

Table 3 presents the descriptive statistics of the homozygous regions. The average number and mean length of extended homozygous regions were minimal in the investigated samples of the Ukrainian Muffed breed. On the contrary, the Faverolles breed showed the highest mean length and mean number of ROHs

**Table 3.**
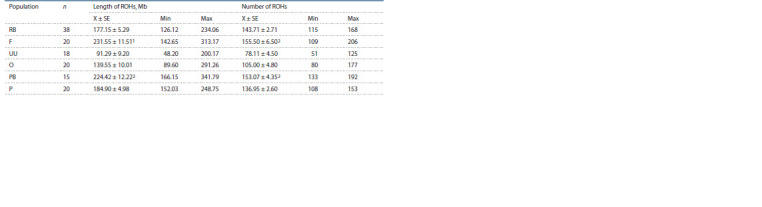
Descriptive statistics of runs of homozygosity determined based on SNP genotypes in the studied chicken breeds RB – Russian White, F – Faverolles, UU – Ukrainian Muffed, O – Orloff, PB – Novopavlov (White population), P – Novopavlov (Coloured population). 1, 2 Differences are not statistically significant. In other cases, differences are significant at a significance level of α = 0.05.

**Fig. 6.**
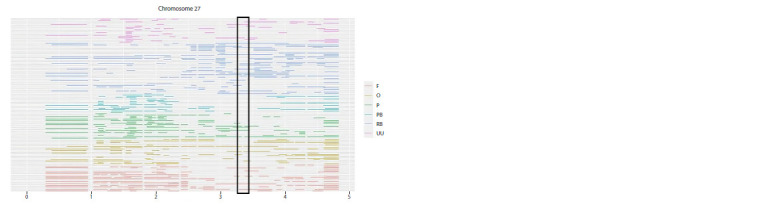
Location of homozygosity runs on chromosome GGA27 in chickens. The highlighted box indicates the region annotated with the Mb allele. RB – Russian White, F – Faverolles, UU – Ukrainian Muffed, O – Orloff, PB – Novopavlov (White population), P – Novopavlov (Coloured population).

As the “muffs and beard” phenotype is linked to the HOM8 gene situated on chromosome GGA27, we investigated the homozygous regions in this genetic segment separately. Figure 6 shows that ROH fragments are infrequent in breeds that exhibit the “muffs and beard” phenotype within the 3.402–3.404 Mb region on GGA27.

## Discussion

Modern methods for studying genomic DNA polymorphism provide extensive data to comprehend the population’s entire genome architecture. Genotyping with chips of different densities allows the phylogenetic divergence of animal breeds to be assessed, and the information obtained can help to maintain distinct genetic diversity of populations (Dementieva et al., 2021; Krivoruchko et al., 2021). A comprehensive understanding of the genetic structures’ specificity is crucial for examining genetic diversity, and it can be applied to explore the historical processes linked to the formation and evolution of populations as separate ecosystems influenced by human beings

In this research, the application of the Chicken 60K Bead- Chip led to the identification of 53,313 single nucleotide polymorphisms. Previous genetic diversity study methods, based on the analysis of mini- and microsatellite loci and mitochondrial DNA, have much lower resolution (Fisinin et al., 2017; Guo H.W. et al., 2017).

The expected (HE) and observed heterozygosity (HO) (see Table 1) derived from the full genomic genotyping data in our study were higher in chickens that emerged as separate clusters in the MDS analysis (see Fig. 3). The initial group, which amalgamated the populations of the Novopavlov breed, exhibited HO value of 0.344, while HE values ranged from 0.338 ± 0.001 to 0.342 ± 0.001. In the second group, comprised of the Ukrainian Muffed and Orloff breeds, the values for HO were between 0.355 ± 0.006 and 0.365 ± 0.005, and those for HE varied between 0.344 ± 0.001 and 0.364 ± 0.001 (see Table 1). These findings concurred with other researchers’ material (Strillacci et al., 2017; Yuan et al., 2022).

Based on the results of the multidimensional scaling analysis, it can be concluded that the breeds most distantly related to each other are the Russian White and Faverolles. The greatest genetic divergence between these breeds lies in their origins. The Russian White chicken breed was developed using white domestic Leghorn chickens as a foundation (Dementeva et al., 2017). Faverolles were bred in France from indigenous chicken breeds. Breeds with similar genetics include Orloff Mille Fleur, Ukrainian Muffed and Faverolles. The White and Coloured populations of the Novopavlov breed are genetically related, as evidenced by their similar architecture. The Ukrainian Muffed breed has similarities to the Orloff Mille Fleur breed in genome fragments. This introgression of genomes between the breeds may have occurred during the late 19th and early 20th centuries, when both breeds flourished and developed in a common area. The utilization of diverse components during the analysis did not alter the spatial pattern of population arrangement, indicating that this method accurately reflects the genuine genetic divergence of breeds.

Assessment of genetic diversity provides greater insight into the genomic structure of chicken breeds and populations (Malomane et al., 2019; Restoux et al., 2022). Pairwise genetic distance FST visualization demonstrated a genetic correlation among the Orloff Mille Fleur, Ukrainian Muffed, and Faverolles breeds, as well as within the White and Coloured populations of the Novopavlov chicken breed (see Fig. 2).

The phylogenetic tree exhibits a conspicuous demarcation of the maximum divergence between the Faverolles and Russian White chicken breeds. The branch that contains the Faverolles, Ukrainian Muffed, and Orloff Mille Fleur breeds reveals their shared ancestry (see Fig. 2). Each mentioned breed forms its own branches in the future. The Orloff Mille Fleur chicken breed is dissimilar from the Ukrainian Muffed type, with contrasting traits and merging represented by the Faverolles chickens. The White and Coloured populations of the Novopavlov breed showed little divergence. Our findings revealed a high level of genetic differentiation between breeds, comparable to literature data described previously in studies of other breeds (Dementieva et al., 2020; Fedorova et al., 2022).

Visualisation of the pairwise genetic distances of the FST using the Neighbourhood Network algorithm, as well as Admixture cluster analysis, confirmed the results of the multidimensional scaling analysis (see Fig. 4). At K = 4, the Orloff and Ukrainian Muffed populations were distinguished from the other populations. In addition, the FST analysis shows that the Ukrainian Muffed population under study has individuals that are genetically similar to the Orloff breed. This may result from the introgression of genomes between breeds. Unintended crossbreeding between Ukrainian Muffed and Orloff populations is possible due to the proximity of the breeding areas of these breeds in the past. At K = 5, the White and Coloured populations of the Novopavlov chicken breed were separated. These findings demonstrate that the populations possess an identical genetic structure, with the Novopavlov White strain having been acquired by selectively breeding white individuals from the coloured population

The high frequency of brief homozygous regions (1–2 Mb) discovered within the Russian White and Ukrainian Muffed breeds suggests that long-term inbreeding has occurred (see Fig. 5). The Russian White chicken breed was obtained through rigorous selection for chick resistance to cold. As a result of a single crossing with White Leghorn in 2005, the Russian White population has no tendency to increase homozygosity, indicating high genetic diversity in the population. Long regions of homozygosity of class 16+ were greater in the Novopavlov chickens (White and Coloured populations), Orloff Mille Fleur, and Ukrainian Muffed breeds, indicating

the presence of recent inbreeding. The Russian White breed has the lowest number of long homozygous regions, an indication that there is no inbreeding within the population due to individual fixation of the producer during breeding (Fedorova et al., 2022; Mulim et al., 2022).

Location analysis of homozygous regions in the region annotated for the HOXB8 gene responsible for the “muffs and beard” phenotype showed the absence of homozygous regions in breeds with this phenotype. This may be attributed to the region’s high variability. Guo’s study (Guo Y. et al., 2016) showed that the presence of the Mb allele causes the HOX8 gene to be ectopically expressed. As a result of the duplication of three regions on chromosome 27, a structural mutation takes place, which does not contribute to the selective accumulation of homozygous regions. ROH regions were discovered on chromosome GGA27, in the range of 1.5–1.6 Mb, occurring in more than 60 % of representatives of all breeds studied, except the Russian White, which lacks muffs and beard (see Fig. 6). Perhaps the buildup of homozygosity in these regions may not be a universal trait for all individuals in the investigated breeds. Consequently, it is plausible to assume that this genome region could serve as a marker for the “muffs and beard” characteristic with a certain degree of probability

## Conclusion

Based on the conducted study, we can state that full genomic genotyping using the Chicken 60K BeadChip (Illumina Inc., USA) medium-density DNA chips is an accurate method for analysing genetic divergence of chicken populations of different historical origins. Similar results can be achieved through a variety of statistical approaches to interpreting data. These results can be explained by considering the historical development of the breed ecosystem as well as the specific method used to detect polymorphism. The gathered data helps us comprehend the particularities of genomic architecture of the studied chicken breeds and populations, and to use this information to control variability in order to preserve genetic diversity. The findings obtained can be used in further research to identify candidate genes for the “muffs and beard” phenotype in chickens, as well as to use gene pool populations as model objects with a high level of genetic diversity

## Conflict of interest

The authors declare no conflict of interest.
